# Right Ventricular Thrombus Presenting With Right Ventricular Failure in Septic Shock: A Case Report

**DOI:** 10.7759/cureus.103900

**Published:** 2026-02-19

**Authors:** Micah Harris

**Affiliations:** 1 Internal Medicine, OhioHealth Riverside Methodist Hospital, Columbus, USA

**Keywords:** diagnostic and therapeutic challenge, interventional cardiology, intracardiac thrombus, pulmonary and critical care medicine, right ventricular cardiac thrombus, right ventricular failure, septic shock (ss)

## Abstract

We present a case report of refractory multifactorial shock initially managed as septic shock, requiring multidisciplinary collaboration when the diagnostic pathway was limited by acute kidney injury; the primary learning objective is to highlight clinical decision-making in shock when standard contrast imaging is contraindicated. Echocardiography revealed right ventricular failure with right ventricular thrombus, and pulmonary embolism was strongly suspected but could not be confirmed due to the inability to obtain contrast imaging. Persistent shock prompted a transition from a septic shock-directed strategy to cardiogenic and obstructive shock management. The patient was treated with anticoagulation but was not a candidate for mechanical thrombectomy. The course was complicated by progressive renal failure, and after dialysis was declined by the family, the patient was transitioned to comfort care.

## Introduction

Sepsis is one of the leading causes of death in the critically ill, and about 15% of patients with sepsis develop a shock state [1]. Prompt management is crucial, as illustrated in recent evidence for early diagnostic cultures, antibiotic therapy, resuscitation, and pressors if required [2]. However, processes are often multifactorial, and it is imperative to constantly evaluate for other possible causes of shock. Persistent shock without improvement despite appropriate sepsis management should prompt reevaluation. Right heart failure can often be seen in septic cases, likely due to a combination of depressed myocardial contractility, increased pulmonary vascular resistance, and inflammatory-mediated myocardial dysfunction, and can contribute to short-term and long-term mortality [3,4].

Right-sided cardiac thrombus, including right ventricular thrombus, is uncommon and may be associated with serious complications such as pulmonary embolism and hemodynamic collapse, but clinical data are limited, and optimal diagnostic and management pathways remain poorly defined [5]. In addition, guidance on shock reassessment is sparse when contrast-based imaging cannot be performed. Our case illustrates the complicated picture of mixed septic and suspected cardiogenic and obstructive shock in the setting of suspected massive pulmonary embolism while highlighting real-world limitations of diagnostic workup, such as acute renal failure preventing the completion of contrasted imaging. The objective of this case report is to demonstrate a structured reassessment approach and management decision-making when persistent shock and imaging limitations complicate the diagnostic pathway.

## Case presentation

A 78-year-old woman with a history of coronary artery disease status post percutaneous coronary intervention to the left anterior descending artery, type 2 diabetes mellitus, hypertension, hyperlipidemia, and obesity presented with one day of altered mental status.

Initial evaluation at the referring hospital revealed a urinalysis concerning for infection, elevated creatinine consistent with acute kidney injury, elevated troponin (145 ng/L, repeat 159 ng/L), and markedly elevated BNP (>70,000 pg/mL). Initial electrocardiogram (ECG) demonstrated normal sinus rhythm with a heart rate of 65 bpm and normal axis without acute ischemic changes. Computed tomography (CT) of the abdomen showed diffuse pancreatic edema. Blood cultures were obtained, and empiric broad-spectrum antibiotics were initiated. Due to concern for volume overload in the setting of markedly elevated BNP, the patient received one liter of crystalloid resuscitation. She required vasopressor support and was transferred to a tertiary hospital for further intensive management.

On arrival at the tertiary center, the patient was receiving norepinephrine at 5 mcg/min. Initial lactate following transfer was 3.2 mmol/L, which peaked at 3.7 mmol/L on hospital day 2 and normalized later that day. Vasopressor requirements increased to a peak norepinephrine dose of 22 mcg/min on day 2, followed by gradual down-titration over the next three days, though a persistent requirement of 2 mcg/min remained until transition to comfort care.

**Figure 1:**
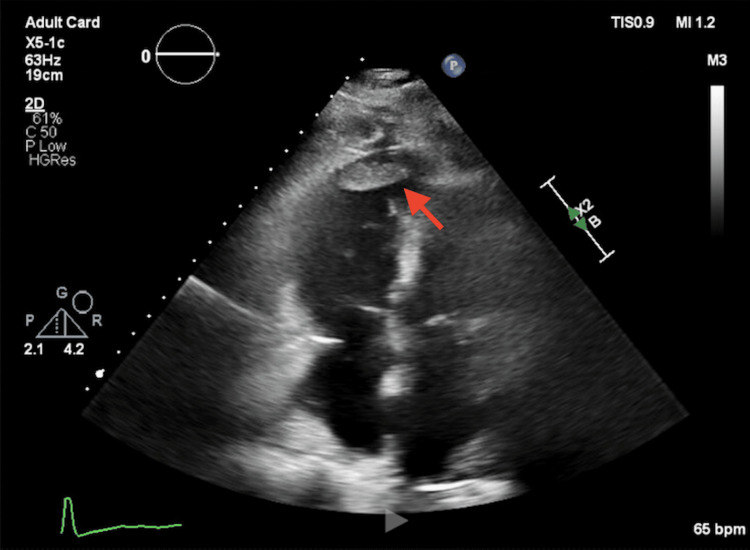
Transthoracic echocardiography showing right ventricular thrombus Transthoracic echocardiography (apical four-chamber view) demonstrates a 4.2×1.1 cm thrombus in the right ventricle (red arrow) with severely reduced right ventricular systolic function.

Broad-spectrum antibiotics were continued for suspected sepsis with possible contribution from pancreatitis. Transthoracic echocardiogram demonstrated normal left ventricular systolic function, but severely reduced right ventricular systolic function and a large right ventricular thrombus measuring 4.2×1.1 cm (Figure 1). A weight-based heparin infusion was initiated immediately. Given these findings, there was great concern for extensive pulmonary embolic disease; however, contrast-enhanced imaging was deferred due to worsening kidney injury. Bedside echocardiography was not repeated because the formal echocardiogram clearly demonstrated right ventricular thrombus and severe dysfunction.

Additional fluids were administered cautiously, and the heart failure service was consulted. The heart failure service attributed the right ventricular dysfunction primarily to sepsis-induced cardiomyopathy rather than acute pulmonary embolism. Urine cultures later grew *Proteus *species, and antibiotics were de-escalated appropriately. Duplex ultrasound of the lower extremities demonstrated extensive deep venous thrombosis, prompting consultation with interventional cardiology. Although significant pulmonary emboli were suspected, the patient was not a candidate for mechanical thrombectomy due to high procedural risk and anticipated need for extracorporeal membrane oxygenation if decompensation occurred. Systemic thrombolysis was considered but deferred given high bleeding risk and relatively stable hemodynamics with ongoing vasopressor support.

Despite appropriate antimicrobial therapy, the patient's course was marked by persistent vasopressor requirements and progressive renal failure. Her shock state was ultimately attributed to new severe right ventricular failure. Nephrology determined she was not a candidate for renal replacement therapy. In the setting of worsening multiorgan failure, palliative care was consulted for goals-of-care discussions, and the patient was transitioned to hospice on hospital day 11.

## Discussion

This case illustrates the difficulty in managing multifactorial shock in the setting of significant diagnostic limitations due to prohibiting comorbidities such as acute renal failure. The patient was appropriately evaluated for suspected septic shock, with supporting evidence for an infectious source related to the urinary tract. Sepsis management includes early blood cultures, prompt antibiotic administration, fluid resuscitation, and vasopressor support when indicated [2]. However, persistent shock despite appropriate antimicrobial therapy necessitated reevaluation for alternative and concurrent etiologies.

Clinical findings supported contributions from septic, cardiogenic, and obstructive shock. Septic shock was suggested by an identified infectious source and partial laboratory improvement following antibiotic therapy. Cardiogenic shock was supported by severe right ventricular systolic dysfunction on echocardiography, markedly elevated BNP, intolerance of aggressive fluid resuscitation, and persistent vasopressor requirements. Obstructive shock was suspected based on the presence of a large right ventricular thrombus and concern for pulmonary embolic disease.

Fluid management was judicious following the discovery of right ventricular failure on echocardiogram. Management of acute right heart failure is complex, as optimization often necessitates a restrictive fluid strategy to avoid worsening right ventricular dilation and interventricular septal shift, in contrast to the aggressive resuscitation typically employed in septic shock [6,7]. The presence of a right ventricular thrombus further complicated the clinical picture by raising suspicion for pulmonary embolism as an additional contributor to shock.

When contrast-enhanced imaging is contraindicated, management of suspected pulmonary embolism may rely on clinical risk stratification, ventilation/perfusion (V/Q) scanning, echocardiography, and lower extremity duplex ultrasonography [8]. In this case, V/Q scanning was not pursued due to the patient's clinical instability and limited ability to tolerate additional imaging. Echocardiographic findings of right ventricular dilation, dysfunction, and intracardiac thrombus are strong indicators of embolic disease and are frequently associated with hypercoagulable states [9]. In high-risk patients, echocardiographic evidence of right ventricular dysfunction can be a useful diagnostic marker in massive pulmonary embolism, although findings may be absent when the clot burden is low [10].

The right ventricular thrombus was felt to most likely represent thrombus-in-transit, given the presence of extensive venous thrombosis and high clinical suspicion for pulmonary embolic disease. The coexistence of right ventricular thrombus and pulmonary embolism has been associated with reduced survival, underscoring the importance of heightened clinical awareness and prompt risk stratification [11]. Contrast-enhanced imaging remains a cornerstone of pulmonary embolism diagnosis, yet the risk of worsening kidney injury significantly limits its use in patients with acute renal failure [12]. In this case, diagnostic decision-making was further complicated by the high risk of contrast-induced nephropathy; the patient's hemodynamic instability and new-onset heart failure made her a poor candidate for hemodialysis should renal recovery not occur.

Given evidence of adequate antimicrobial coverage, absence of persistent fevers, and improving leukocytosis in the setting of continued low-dose vasopressor dependence, attention shifted toward the definitive management of suspected cardiogenic shock secondary to right ventricular failure and obstructive shock secondary to pulmonary embolism. Both interventional and medical management options were limited by the patient's high risk for decompensation. Although systemic thrombolytic therapy has been shown to reduce mortality in high-risk pulmonary embolism, it carries a substantial risk of major hemorrhage, including fatal and intracranial bleeding [13]. In this context, thrombolysis was deemed prohibitively high risk due to the patient's decompensated heart failure and inability to tolerate renal replacement therapy. Consequently, goals-of-care discussions were initiated, ultimately resulting in a transition to hospice.

## Conclusions

This case demonstrates the importance of continual reassessment in patients with multifactorial shock, particularly when standard imaging is contraindicated due to comorbidities such as acute renal failure. When contrast studies cannot be performed, echocardiography and bedside clinical evaluation can provide critical diagnostic information to guide risk-stratified management decisions. Persistent shock despite appropriate infectious management should prompt clinicians to consider concurrent cardiogenic or obstructive etiologies and to prioritize interventions that balance potential benefit against patient-specific risks. The approach illustrated in this case, using targeted diagnostics and carefully titrated therapies while awaiting confirmatory imaging or when imaging is impossible, highlights how clinicians can operationalize a risk-guided, patient-centered management strategy. These observations are intended to inform clinical reasoning and highlight challenges in complex cases, rather than dictate standard-of-care protocols.
